# Review of "*Spatial Analysis in Epidemiology*" by D.U. Pfeiffer, T.P. Robinson, M. Stevenson, K. B. Stevens, D.J. Rogers and A.C.A. Clements

**DOI:** 10.1186/1756-3305-2-23

**Published:** 2009-04-29

**Authors:** María-Gloria Basáñez

**Affiliations:** 1 Department of Infectious Disease Epidemiology, Faculty of Medicine (St Mary's campus), Imperial College London, Norfolk Place, London, W2 1PG, UK

## Review

The consideration of the need for, and application of, spatially-explicit analysis in chronic and infectious disease epidemiology and control has been gaining momentum, particularly over the last two decades. In part this is because of an increased awareness of the importance of interconnectedness of individuals and populations, and in part because of increased availability of technologies for obtaining, visualising and analysing geographically explicit information. The development of spatial statistics has prospered from this momentum and has expanded from the realm of theoreticians to provide tools in the armamentarium of ecologists, parasitologists, epidemiologists, and infectious disease biologists among others. It is to the students and practitioners of these disciplines that this book is aimed, and it constitutes an excellent introduction to the field for the uninitiated. The book also provides a glossary of the many abbreviations used in spatial statistics and analysis, a source of useful websites and other resources, and a comprehensive and up to date reference list. The layout is very attractive as the book is well and didactically structured, with a section of concluding remarks at the end of each chapter that helps to summarise the 'take home' messages. A particularly good feature is the use of a consistent dataset wherever possible (data from 1986 to 1999 for bovine tuberculosis arising from the UK's national control programme) to illustrate the various techniques and analyses discussed. After an introductory chapter, laying the scene and describing the book's aims and structure, chapter 2 deals with the nature of spatial data; chapter 3 with issues and methods for spatial visualization and representation of such data; chapters 4, 5 and 6 with spatio-temporal clustering and variation of disease risk at global and local levels (exploratory analyses), and chapters 7 and 8 focus on modelling techniques for assessment of risk factors and policy decision support. The book is multi-authored but obviously carefully edited for consistency throughout the text though not necessarily so for notation, which sometimes differs between text and equations. Some techniques are more clearly explained than others, again possibly a consequence of different authors contributing to various sections. Having said this, I found that the book provides a very useful introduction to spatial analysis in epidemiology and, I am sure, a reference that I will often revisit.

## Competing interests

The author declares that they have no competing interests.

